# Alpha-lipoic acid treatment is neurorestorative and promotes functional recovery after stroke in rats

**DOI:** 10.1186/s13041-015-0101-6

**Published:** 2015-02-11

**Authors:** Kang-Ho Choi, Man-Seok Park, Hyung-Seok Kim, Kyung-Tae Kim, Hyeon-Sik Kim, Joon-Tae Kim, Byeong-Chae Kim, Myeong-Kyu Kim, Jong-Tae Park, Ki-Hyun Cho

**Affiliations:** Department of Neurology, Chonnam National University Hwasun Hospital, Hwasun, Korea; Department of Neurology, Chonnam National University Medical School, 8 Hak-dong, Dong-gu Gwangju, 501-757 Korea; Department of Forensic medicine, Chonnam National University Medical School, 8 Hak-dong, Dong-gu Gwangju, 501-757 Korea; Department of Anesthesiology and Pain Medicine, Inje University Ilsan Paik Hospital, Goyang, Korea; Department of Nuclear Medicine, Chonnam National University Hwasun Hospital, Hwasun, Korea

**Keywords:** Stroke, Antioxidants, Neuroprotection, Neurorestoration, Acute therapy

## Abstract

The antioxidant properties of alpha-lipoic acid (aLA) correlate with its ability to promote neuroproliferation. However, there have been no comprehensive studies examining the neurorestorative effects of aLA administration after the onset of ischemia. The middle cerebral artery (MCA) of adult rats was occluded for 2 hours and then reperfused. aLA (20 mg/kg) was administered in 71 animals (aLA group) through the left external jugular vein immediately after reperfusion. An equivalent volume of vehicle was administered to 71 animals (control group). Functional outcome, levels of endogenous neural precursors with neurogenesis, glial cell activation, and brain metabolism were evaluated. Immediate aLA administration after reperfusion resulted in significantly reduced mortality, infarct size, and neurological deficit score (NDS) in the test group compared to the control group. Long-term functional outcomes, measured by the rotarod test, were markedly improved by aLA treatment. There was a significant increase in the number of cells expressing nestin and GFAP in the boundary zone and infarct core regions after aLA treatment. Furthermore, significantly more BrdU/GFAP, BrdU/DCX, and BrdU/NeuN double-labeled cells were observed along the boundary zone of the aLA group on days 7, 14, and 28 days, respectively. And brain metabolism using ^18^F-FDG microPET imaging was markedly improved in aLA group. The effects of aLA was blocked by insulin receptor inhibitor, HNMPA (AM)3. These results indicate that immediate treatment with aLA after ischemic injury may have significant neurorestorative effects mediated at least partially via insulin receptor activation. Thus, aLA may be useful for the treatment of acute ischemic stroke.

## Introduction

Stroke is a leading cause of death worldwide and the largest single cause of long-term disability in millions of people in developed countries [1]. In particular, large hemispheric infarctions due to middle cerebral artery occlusion (MCAO) are a major cause of severe morbidity and mortality. Despite sophisticated medical management and neurosurgical techniques, middle cerebral artery (MCA) territory infarction still results in a high mortality rate of 40% to 80% [2]. A better understanding of the major factors contributing to cerebral ischemic damage would improve the functional outcomes in cerebral ischemia and significantly influence the clinical management and prognosis of stroke.

There is a considerable body of evidence to suggest that oxidative stress is a fundamental mechanism causing brain damage in stroke and reperfusion ensuing stroke [3]. The brain is highly susceptible to reactive oxygen species (ROS) that induce damage due to low levels of protective antioxidants, high concentrations of peroxidizable lipids, high oxygen consumption, and high iron levels that act as pro-oxidants under pathological conditions [4]. Therefore, ROS have been implicated as one of the earliest and most important components of tissue injury after reperfusion of an ischemic organ [5].

Alpha-lipoic acid (aLA) is an antioxidant commonly used for treatment of many neurological disorders such as diabetic polyneuropathy and multiple sclerosis. It is absorbed from the diet and crosses the blood–brain barrier [6]. It is not toxic at therapeutic doses [7]. This strong antioxidant influences a number of cellular processes, including direct radical scavenging, recycling, metal chelation, regeneration of endogenous antioxidants, and modulation of transcription factor activity. It has been shown to improve endothelial function and blood flow, and accelerate glutathione synthesis, which plays a crucial role in regulating the expression of several antioxidant and anti-inflammatory genes [6,8-10].

The neuroprotective effects of aLA pretreatment for ischemic injury have been shown in several different experimental models of MCAO in rodents [11-15]. Recent evidence from an *in vitro* experimental model suggests that the antioxidant properties of aLA, specifically its ability to restore glutathione content, correlate with its ability to promote glial-neuronal interactions [16]. Previous studies in this area are limited and have either (a) focused on one specific antioxidant mechanism rather than a more comprehensive analysis, (b) utilized pretreatment with aLA rather than administration after the onset of ischemia, or (c) evaluated only early ischemic injury rather than the long-term effects of treatment.

To date there have been no reports of comprehensive studies examining the long-term effects and outcomes of aLA administered after the onset of ischemia. Using a rat model that closely approximates clinical stroke with MCAO, we sought to investigate the long-term neurorestorative effects of aLA in immediate treatment after cerebral ischemic injury, particularly those related to neuroproliferation.

## Materials and methods

### Animal preparation

All animal procedures were performed in accordance with the Chonnam National University guidelines for the care and use of laboratory animals and were approved by the Institutional Animal Care and Use Committee (IACUC) of Chonnam National University (Permit Number: 2011–14). All experiments were carried out in accordance with the guidelines laid down by the National Institutes of Health (NIH, USA) regarding the care and use of animals for experimental procedures. Adult (8-week-old) male Sprague–Dawley (SD) rats weighing 253–288 g (n = 201) were purchased from Samtako (Seoul, Korea).

To examine the effect of aLA on brain infarction, aLA injection (20 mg/kg body weight [B.W.], Bukwang pharmaceutic company, Seoul, Korea), was administered immediately after reperfusion of the left MCAO through the left external jugular vein in 71 animals (aLA group, Table 1). An equivalent volume of vehicle (0.9% NaCl) was administrated in 71 animals (control group) using the same procedure. As an additional control for non-specific findings, 10 animals underwent sham surgery (sham group). In the sham group, 10 animals were used for behavioral test. Among them, 6 animals were used for determination of infarct volume and molecular studies at scheduled time points.

**Table 1:** **Total numbers and groups of Sprague–Dawley rats used in this study**

	**aLA group**	**Control group**	**HNMPA group**	**Sham group**
Age (wk)	8.0 ± 0.3	8.0 ± 0.3	8.0 ± 0.3	8.0 ± 0.3
Body weight (g)	270.9 ± 8.3	271.3 ± 8.1	270.8 ± 6.1	271.1 ± 7.5
No. of animals used for main experiment	71	71	49	10
Micro-PET image/TTC stain	1/30 (10 rats/day)	1/30 (10 rats/day)	0/30 (10 rats/day)	0/3 (1 rat/day)
Behavior test	20	20	10	10
Micro-PET image/IHC & RT-PCR	1/9 (3 rats/day)	1/9 (3 rats/day)	0/9 (3 rats/day)	0/3 (1 rat/day)
Immunofluorescence	12 (4 rats/day)	12 (4 rats/day)	0	0
Mortality, %	29.6	53.5	46.9	0

In the previous study, hydroxy-2-naphthalenylmethyl phosphoric acid triacetoxymethyl ester (HNMPA(AM)3), a highly specific insulin receptor (IR) inhibitor, blocked the protective effects of aLA in hepatocyte [17,18]. Therefore, to further verify the effect of aLA, HNMPA(AM)3 (200 uM, Santa Cruz Biotechnology, Santa Cruz, CA, USA) was pretreated for 3 h before aLA treatment in 49 animals (HNMPA group).

### Surgical procedures

The left MCA of 191 SD rats was occluded for 2 hours using the intraluminal filament technique, as previously described [19]. Rats were anesthetized with isoflurane (3% for induction and 2% for maintenance) in a mixture of oxygen/nitrous oxide (30/70). Their body temperature was maintained at 36.6 ± 0.5°C throughout and following surgery with a thermostatically controlled heating pad. All efforts were made to minimize suffering. After a 2-hour occlusion, reperfusion was performed as described previously. Sham surgery was carried out by introducing the thread through the left MCA, followed by immediate withdrawal in 10 SD rats. All other procedures in the sham group were identical to the ischemic surgery groups.

Rats were placed in cages with normal water and food after reperfusion. Mortality rate of the rats as a direct result of ischemic stroke was calculated and compared after reperfusion. The MCA occluded rats were observed closely for the first 2 hours, hourly for the next 6 hours, thereafter four times a day for 7 days, and twice a day after the first week. Animals were humanely sacrificed by cervical dislocation after anesthesia by an expert at 1, 3, 7, and 14 days following MCAO for the measurement of cerebral infarct volume and immunostaining analysis. At each time point, rats were anesthetized with isoflurane and the brains were removed quickly. Rats that survived until the final endpoint for behavioral testing and survival analysis were humanely euthanized with an overdose of sodium pentobarbital. when they met the euthanasia guidelines of Chonnam National University, signs of severe central nervous system dysfunction non-responsive to treatment.

### BrdU administration

**Figure 1:**
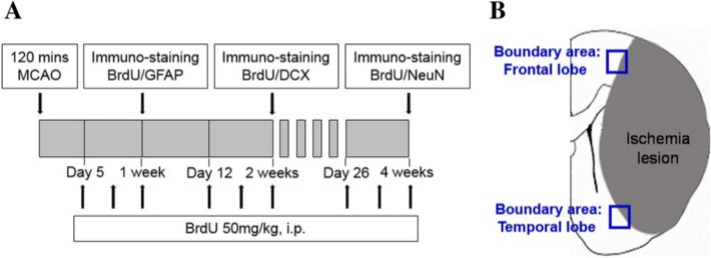
**Overview of double immunostaining design. (A)** Diagram of the initial experiments comparing early proliferation of astrocytes (BrdU/GFAP-positive) at 1 week and mature neuron formation at 4 weeks (BrdU/NeuN-positive) after middle cerebral artery occlusion (MCAO). **(B)** Coronal sections delineating the main areas of interest: frontal and temporal boundary zone.

The thymidine analog 5-bromo-2′-deoxyuridine (BrdU; Sigma-Aldrich, St Louis, MO, USA) was used to label proliferating cells. BrdU was dissolved in 0.9% sterile NaCl and filtered at 22 μm. The resulting solution was injected at 50 mg/kg (10 mg/mL) intraperitoneally in both groups. Injections were given 5 days after ischemic insult for 3 days, a period that is documented to be during peak cell proliferation after ischemia [20], and additionally given once daily for 3 days before rats were euthanized (2 weeks and 4 weeks post-MCAO) to maximize the labeling of proliferating cells(Figure 1).

### Determination of infarct volume

Ten rats from the aLA and control groups were euthanized by deep isoflurane anesthesia at 1, 3, and 7 days after occlusion. The brains were carefully removed from the skull and then sectioned in the coronal plane at 2 mm intervals, starting at 1 mm from the frontal pole, using a rodent brain matrix (RBM-4000C; ASI Instruments, Warrin, MI, USA). Coronal slices were incubated in phosphate-buffered saline (PBS) containing 2% 2,3,5-triphenyltetrazolium chloride (TTC; Sigma) at room temperature for 1 hour and then transferred to 4% buffered paraformaldehyde solution for fixation [21]. The area of brain damage over the entire ipsilateral hemisphere was measured using the Adobe Photoshop software (Adobe Systems) and the total volume of infarction (mm^3^) was calculated from the sum of the damaged volumes (i.e., damaged areas × thickness) in the brain slices.

### ^18^F-FDG positron emission tomography (PET) imaging

Regional glucose metabolism of the brain lesion was evaluated *in vivo* by ^18^F-FDG microPET imaging 1 day and 7 days after cerebral MCAO on a microPET R4 scanner (Concorde Microsystems, Knoxville, TN). Under brief (2 min) halothane gas anesthesia, the rats were intravenously injected with 2 mCi/mmol ^18^F-FDG through the tail vein. The animals were then returned to their cage and placed in a room with minimal ambient noise. After an uptake period of 60 min, the animals were placed in the microPET scanner under halothane gas anesthesia (5% induction and 1.5% for maintenance). A 20 min static acquisition was performed in 3-D mode. Data were collected in list mode and reconstructed by a maximum a-posteriori probability algorithm with a pixel size of 0.4 × 0.4 × 1.2 mm^3^.

The imaging data acquired from the microPET were displayed and analyzed by IDL (ver. 6.2, Research Systems, Colorado, USA). Axial MR images were used to assign regions of interest (ROIs) per slice according to a rat brain atlas. Glucose metabolism was evaluated by drawing ROIs in the bilateral cortex and striatum for each slice. A semiquantitative method (standardized uptake value, SUV) was used to calculate the changes in metabolic rate of the bilateral cortex and striatum.

### Behavioral tests of ischemic stroke model rats

All animals were trained for neurobehavioral assessment for 7 days before MCAO. A neurological examination was performed at defined points after MCAO. An investigator who was blinded to the experimental groups determined the neurological deficit score (NDS) based on (a) consciousness: 0, normal; 1, restless; 2, lethargic; 3, stuporous, (b) gait: 0, normal; 1, paw adduction; 2, unbalanced walking; 3, circling; 4, unable to stand; 5, no movement, (c) limb tone: 0, normal; 1, spastic; 2, flaccid, and (d) pain reflex: 0, normal; 2, hypoactive; 4, absent, at 2 hours and 1, 3, 7, 14, 21, 28, 35, 42, 49, and 56 days after MCAO by [21]. A higher NDS indicated a worse neurological status.

Motor impairment was assessed using the accelerating rotarod test. A 5 min activity test was performed for each experimental group. Prior to the induction of ischemia, rats were trained on the rotarod for five consecutive days, for 15 sessions in total. The rotating drum was accelerated from 4 to 40 rpm over 5 min and the time taken (in seconds) for the animal to fall off the drum was recorded. Each session included five consecutive trials with a maximum time of 300 s; the mean latency to fall was calculated from the five trials. Animals that could not stay on the rod for an average of at least 250 s by the end of training were excluded from the stroke surgery.

Spontaneous motor activity (SMA) was evaluated for 5 min by placing the animals in their normal environment (their cage). Neurological scoring of SMA was assigned as follows: 0, moved about the cage and explored the environment; 1, moved about the cage but did not approach all sides and was hesitant to move; 2, barely moved about the cage and showed postural abnormalities (curved towards the paretic side); 3, unable to move and posture curved towards the paretic side. The flexion test was scored as follows: 0, no observed neurological deficit; 1, contralateral forelimb flexion with wrist flexion and shoulder adduction; 2, reduced resistance to lateral push; 3, circling movements towards the paretic side [22,23]. Behavioral testing was conducted at 3, 7, 14, 21, 28, 35, 42, 49, and 56 days after surgery.

### Immunohistochemistry

Paraffin-embedded sections were deparaffinized, rehydrated successively in a gradient of ethanol, and then treated twice in a pressure cooker for antigen retrieval. After rinsing in PBS 3 times for 2 min each, the sections were incubated in IHC blocking solution (Millipore, Billerica, MA, USA) for 20 min at room temperature to block non-specific binding sites. They were then incubated with anti-rabbit polyclonal glial fibrillary acidic protein (GFAP) antibody (Millipore, Billerica, MA, USA, 1:1,000) or anti-mouse monoclonal nestin antibody (Millipore, Billerica, MA, USA; 1:200) for 2 hours at 37°C, and finally, with biotinylated anti-mouse and -rabbit secondary antibody (IgG) for 30 min at 37°C. Sections were washed 4 times with PBS for 5 min each and then covered with water-soluble mounting reagent. The immunoreactivity was visualized by treating with 0.02% 3,3′-diaminobenzidine (DAB). To prevent variations in DAB staining, we processed all tissue in a single batch.

Paired samples from one aLA-treated and vehicle-treated rat were immunostained and two fields (magnification, 40×, 200×, and 400×) in the frontal and temporal cortex were captured using an Olympus BX 50 optical microscope (Olympus Optical Co., GmbH, Hamburg, Germany). GFAP and nestin immunoreactivity in aLA and control rats at each time point after surgery were analyzed. Immunostained samples were captured with a Nikon camera (Digital Sight DS, Kanagawa, Japan) equipped with Nikon NIS-Elements imaging software (Nikon, Melville, NY).

### Immunofluorescence

To determine levels of neurogenesis, double labeling was carried out with fluorescent-tagged secondary antibodies. After rinsing with washing buffer (PBS with 1% BSA and 0.1% Tween 20), we incubated sections in the appropriate primary antibodies against BrdU (Accurate Chemical and Scientific Corp., Westbury, NY, USA; 1:200 anti-mouse) as a marker of newborn cells, GFAP (DAKO Corporation, Via Real, Carpinteria, CA, USA; 1:500 anti-rabbit) for astrocytes, doublecortin (DCX; Santa Cruz Biotechnology, Santa Cruz, CA, USA; 1:100 anti-goat) for neuronal progenitor cells at 2 weeks, or NeuN (Chemicon International, Temecula, CA, USA; 1:200 anti-rabbit), for mature neurons at 4 weeks (Figure 1). Cells were counterstained with Alexa 488 donkey anti-rabbit (1:200; Invitrogen) and Cy3 donkey anti-mouse (1:200; Jackson ImmunoResearch Europe Ltd., Suffolk, UK) for 60 min at room temperature. The slides were mounted with Vectashield mounting medium containing 4′-6-Diamidino-2-phenylindole (DAPI; Vector Laboratories Inc., Burlingame, CA, USA) and examined in a Leica TCS SP confocal microscope (Leica Microsystems Gmbh, Wetzlar, Germany). Images were analyzed with Adobe Photoshop version 5.5 (Adobe systems, San Jose, CA, USA). We applied the optical fractionator method to count double-labeled cells (BrdU/GFAP–positive, BrdU/DCX–positive, or BrdU/NeuN–positive cells). Each brain had five contiguous homologous coronal sections containing the infarct between +2.2 and −1.3 mm from the bregma as previously described [24]. In each brain, double-labeled cells were counted along the boundary area of the frontal and temporal cortex (Figure 1) and a mean cell count was obtained. Each brain was analyzed by two independent investigators (WY Choi and JH Jeon) who were blind to the treatment group. Confocal images were taken with the Zeiss LSM-510 microscope and multiple z-sections were obtained to ensure colocalization.

### RNA isolation and reverse transcription polymerase chain reaction (RT-PCR)

RT-PCR was used to analyze the mRNA expression of the inflammatory cytokines tumor necrosis factor-alpha (TNF-α), macrophage inflammatory protein 1 (MIP1), ionized calcium binding adaptor molecule 1 (Iba-1), and interleukin-1 beta (IL-1β). In addition, RT-PCR analysis for SOX2, a transcription factor, and nestin, neuronal precursor cells, was performed at 3, 7, and 14 days after MCAO. Total RNA was isolated from the brain by using Trizol (Life Technologies, NY, USA) and then converted to cDNA using a high capacity cDNA reverse transcription kit (Life Technologies). Glyceraldehyde-3-phosphate dehydrogenase (GAPDH) was used as an internal control. RT-PCR was performed using the 7900 HT fast real-time PCR system and primers (Life Technologies). For all RT-PCR plates, samples were run in triplicate to eliminate potential errors and variance between wells. The results of the real-time PCR data are presented as Ct values.

### Statistical analysis

All data are expressed as mean ± standard error of the mean (SEM). A Student’s *t* test or the Mann–Whitney-*U* test was used to compare the results of TTC staining between the aLA and control groups. Continuous variables were compared between the aLA, control, and sham groups by the Kruskal-Wallis test. Statistical comparisons were performed using ANOVA with repeated measures to assess behavioral performance. Survival analysis was used to compare mortality between the aLA and control groups. A *P* value of <0.05 was considered statistically significant. All measurements were taken by observers blind to the individual treatments. All statistical analyses were performed using PASW 18.0 for Windows (SPSS, Inc., Somers, NY, USA).

## Results

### The volume of damage assessed by TTC staining

**Figure 2:**
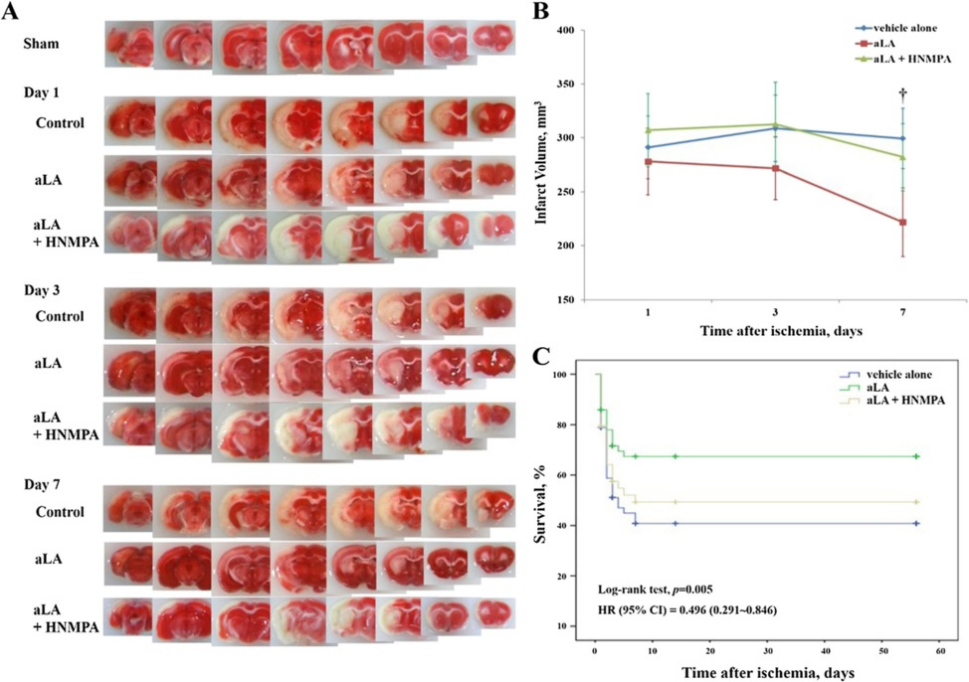
**Changes in infarct volume and mortality. (A)** 2% 2,3,5-triphenyltetrazolium chloride staining of ischemic infarct regions in alpha-lipoic acid (aLA) and control animals following artificial occlusion of the left MCA at 1, 3, and 7 days after the occlusion. **(B)** Infarct volume was quantified and compared between the three groups. Infarct volume significantly decreased in the aLA group 7 days after occlusion (†*P* < 0.01 as compared to the control; Student’s unpaired *t* test). Data represents mean ± SEM (n = 10 rats/day in aLA, control, and HNMPA groups, n = 1 rat/day in the sham group). **(C)** Kaplan–Meier survival curves for aLA (n = 71), control (vehicle alone, n = 71), and HNMPA (n = 49) groups over 56 days following left MCAO.

TTC staining of brain sections was obtained from MCAO rats. It showed reproducible and readily detectable lesions in the areas supplied by the MCA at 1, 3, and 7 days after reperfusion (Figure 2A). The lesions were present in the hippocampus, lateral striatum, and the overlying cortex. As shown in Figure 1, infarct volume in the group of ischemic rats treated with aLA (aLA group) was decreased at 1, 3, and 7 days after left MCA occlusion when compared with the group of rats treated with vehicle alone (control group). Infarct volume was significantly reduced at 7 days by aLA treatment when compared with the control group. The volume of damage at 7 days was approximately 26.0% lower in the aLA group (221.8 ± 31.5 mm^3^, *P =* 0.002) than the control group (299.4 ± 21.1 mm^3^; Figure 2B). This effect was blocked by pretreatment of HNMPA(AM)3, a selective insulin receptor inhibitor. The volume of cerebral infarction at 7 days was 282.1 ± 31.1 mm^3^ in the HNMPA group. We did not observe cerebral infarction in the sham group.

### Increased survival rate in MCAO rats treated with aLA

At 56 days after reperfusion, 38 of 71 rats who underwent MCAO had died in the control group (53.5% mortality) vs. 21 of 71 rats in the aLA group (29.6% mortality). The mortality rate demonstrated a 24% absolute increase in survival for rats treated with aLA relative to those treated with vehicle alone. The cumulative probability of vascular death was significantly lower for the aLA group than the control group in Kaplan-Meier survival curves (log-rank test, *P* = 0.005; HR = 0.496; CI = 0.291–0.846; Figure 2C). Coadministration of the IR inhibitor HNMPA(AM)3 increased the mortality rate similar to that of control group.

### Regional cerebral metabolic changes related to aLA treatment

**Figure 3:**
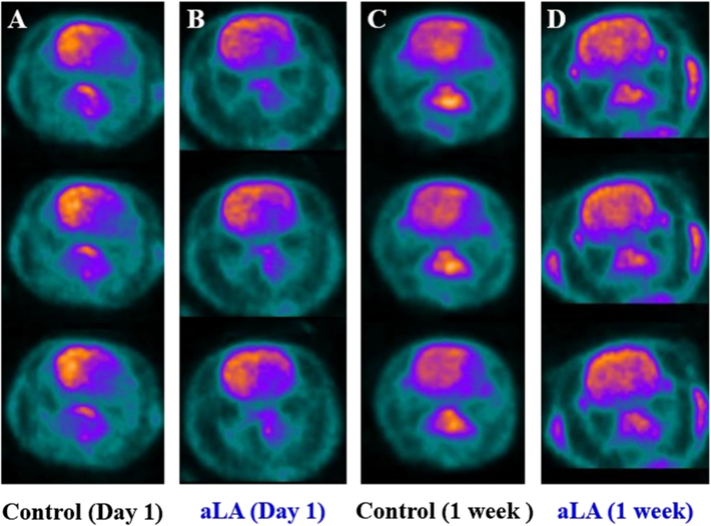
**The effects of aLA treatment on metabolic glucose activity as measured by microPET.** One day after MCAO, decreased uptake of ^18^F-FDG was revealed in the PET images of coronal sections in the rat brains **(A and C)**. By 7 days after MCAO, the hypometabolic region remained distinct in the control group **(B)**, while it lessened significantly in ischemic rats receiving aLA **(D)**.

MCAO decreased metabolic activity in the affected (left) hemisphere by more than 50% than the contralateral hemisphere, as measured by ^18^F-FDG microPET imaging (Figure 3A, B). There was no significant difference between SUVs of the cortex and striatum at 1 day after cerebral ischemia in both groups. However, the SUVs in the cortex and striatum of the affected hemisphere were significantly higher in the aLA group than in the control group at 7 days after cerebral ischemia (*P* < 0.01, Figure 3C, D). The SUVs in the cortex and striatum recovered to 91.2 ± 10.3% of the corresponding homologous contralateral regions in the aLA group, while the values were not recovered when compared with the corresponding contralateral values in the control group at 7 days after cerebral ischemia. The SUVs in the contralateral cortex and striatum were not significantly different in either group at all the times studied.

### Functional recovery

**Figure 4:**
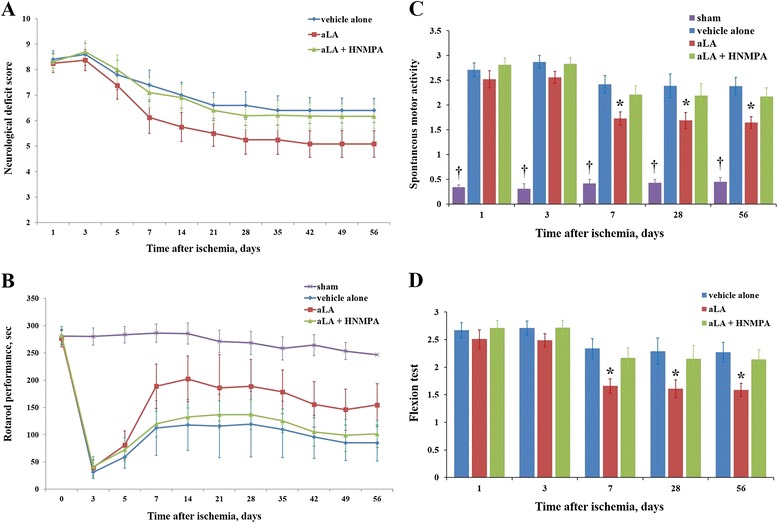
**The effect of alpha-lipoic acid (aLA) on functional recovery. (A)** The initial neurological deficit score (NDS) following ischemic stroke was similar for three groups. After treatment with aLA, the NDS was significantly different, with the lowest scores observed for the aLA group (5.09 ± 0.52) when compared with the control group (6.41 ± 0.48, *P =* 0.001; ANOVA with repeated measures). **(B)** Artificial occlusion of the MCA resulted in severe impairments in the rotarod performance test. Postoperative motor function in the rotarod test was significantly improved by aLA treatment when compared with the control group (*P* = 0.013; ANOVA with repeated measures). **(C)** In the spontaneous motor activity (SMA) test, there was a marked decrease (*P < 0.05; †*P* < 0.01 as compared to the control; Kruskal-Wallis test) in behavioral efficiency in the ischemic group when compared with sham animals. aLA treatment significantly improved behavioral output. **(D)** In the flexion test, there was a marked improvement (*P < 0.05 as compared to the control; Mann–Whitney-*U* test) in the aLA group when compared with the control group. Data represents mean ± SEM (n = 20 in the aLA and control groups, n = 10 in the HNMPA and sham group).

To ensure that all MCAO animals were subjected to similar ischemic insult, the extent of acute neurological deficit by NDS was evaluated in these as well as the sham animals. NDS was significantly greater in MCAO animals than the sham animals, and was similar for both the aLA and control groups (Figure 4A). Initial neurological function was similar for each groups of MCAO animals at 1 day after reperfusion (aLA, control, and HNMPA groups). NDS was significantly lowered in the aLA group (5.09 ± 0.52, *P =* 0.001) than the control group (6.41 ± 0.48). HNMPA blocked aLA-induced functional recovery tested by NDS (6.27 ± 0.79, Figure 4A). We did not observe any neurological deficits in sham-operated animals (NDS = 0).

Prior to MCAO, the time spent on the accelerating rotarod was similar in both, the aLA and control groups. In the sham group, there was no evidence of a neurological deficit in the rotarod test, whereas MCAO animals exhibited severe neurological deficits up to 3 days after reperfusion. Remarkably, aLA-treated rats showed significantly improved recovery during the postoperative period when compared with control animals (*P =* 0.013; Figure 4B), while this aLA effect was not observed in the HNMPA group (Figure 4B).

In the SMA test, ischemic animals spent most of their time in the center of the cage, with a curved posture towards the paretic side. There was a marked decrease (*P* < 0.05) in behavioral efficiency in the ischemic group when compared with sham animals. aLA treatment significantly improved SMA; aLA-treated rats moved around the cage and explored their environment more efficiently than control animals. Behavioral output improved markedly (*P* < 0.05) in the aLA group than the control group (Figure 4C). In the flexion test, ischemic animals showed persistent circling movements with significant severe paw flexions. The aLA group exhibited less persistent circling and comparatively less severe paw flexions with decreased postural disturbances (Figure 4D). We found that HNMPA blocks aLA related functional recovery on behavioral test (Figure 4). Neurological score of the flexion test was 0 in the sham group.

### Effect of aLA treatment on histopathological changes in MCAO rats

**Figure 5:**
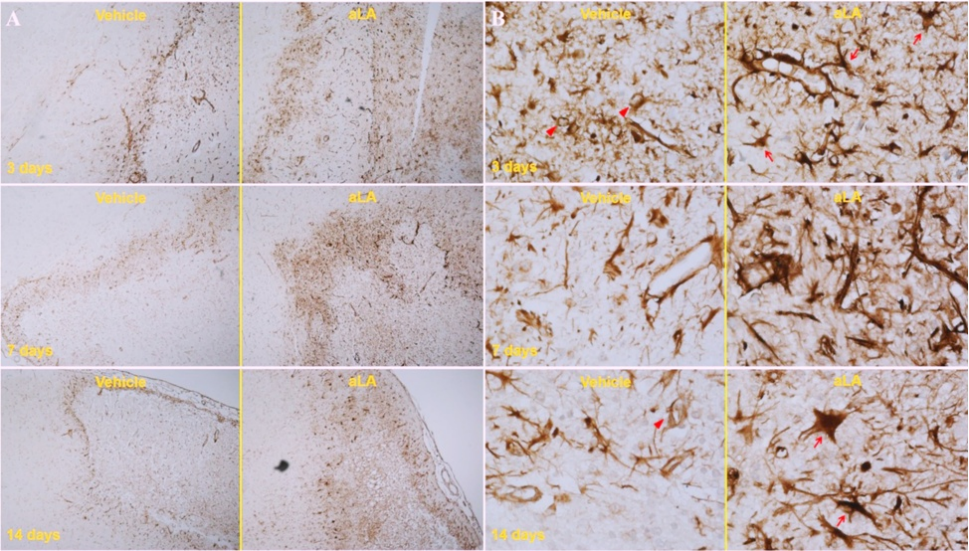
**Immunohistochemical (IHC) staining for anti-nestin in the peri-infarct area at 3, 7, and 14 days after ischemia (magnification, A 40×; B 400×).** Compared with the corresponding regions of the control group, there was an increase in nestin-positive cells in the aLA group at 3, 7, and 14 days after the MCAO. Nestin-positive cells in the aLA group exhibited a variety of morphological features such as stellar shape, large cell body, and multiple processes (arrow). In contrast, stained cells in the control group exhibited small cell bodies, vacuolization, degradation, and loss of processes (arrowhead). n = 3 rats/day/group.

**Figure 6:**
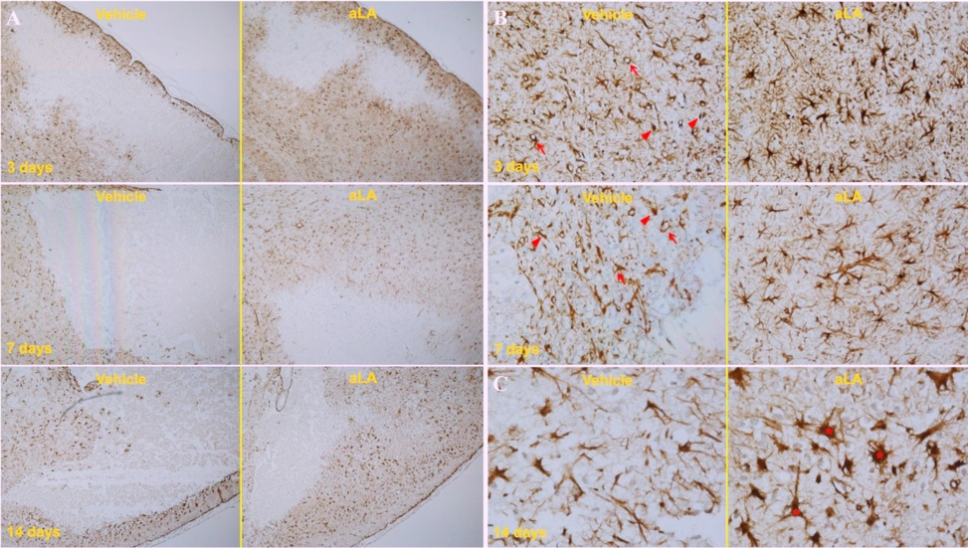
**IHC staining for anti-GFAP in the in the peri-infarct area at 3, 7, and 14 days after ischemia (magnification, A 40×; B 200×; C 400×).** The number of GFAP-positive cells was increased in the aLA group when compared with the control group. In vehicle-treated animals, a greater infarct core region was clearly defined at 7 and 14 days, compared with 3 days. By contrast, the infarct core region was not well defined in aLA-treated animals, because of the presence of cells that had grown inside the infarct core region from the boundary of the lesion at 7 and 14 days. Furthermore, GFAP-positive cells with necrotic morphology, vacuolization (arrow), and loss of processes (arrowhead) in the peri-infarct area were markedly reduced in aLA-treated animals when compared with controls. In contrast, GFAP-positive cells had large cell bodies and multiple processes (star) in the aLA group. n = 3 rats/day/group.

**Figure 7:**
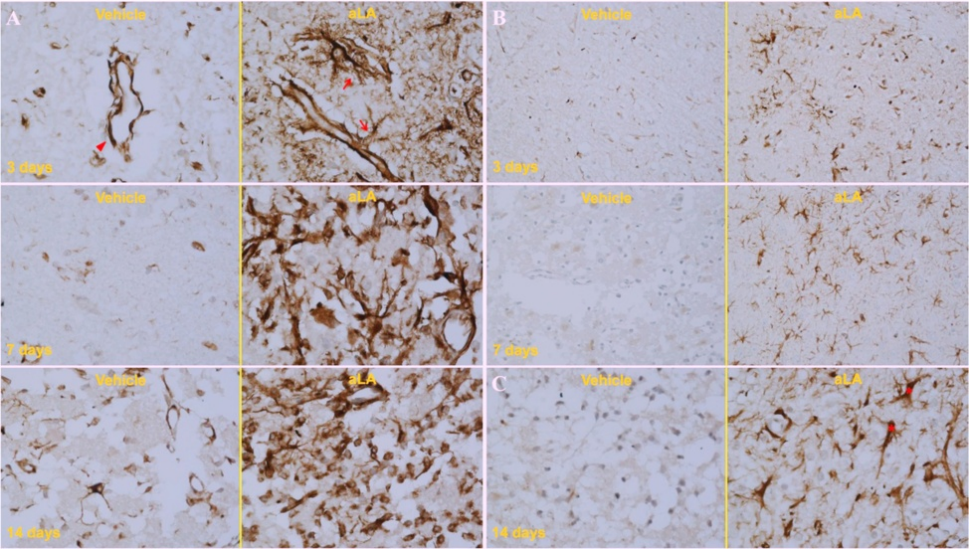
**IHC staining for anti-nestin (A) and GFAP (B, C) protein following ischemia in the infarct core region at 3, 7, and 14 days after ischemia (magnification, A, C 400×; B 200×).** Compared with the corresponding regions in the control group, there was an increase in nestin- and GFAP-positive cells and vessels in the aLA group at 3, 7, and 14 days after the MCAO. Additionally, the morphological features of nestin- and GFAP-expressing cells and vessels, such as the number and shape of cell bodies and processes, were significantly improved in the aLA group (arrow and star). In contrast, the control group exhibited loss of processes (arrowhead). n = 3 rats/day/group.

Figures 5, 6 and 7 show the histopathological changes in the peri-infarct area and infarct core regions at 3, 7, and 14 days after MCA occlusion in aLA and control rats. Sections of the brain passing from the frontal cortex were examined. In the infarct core region of the control group, there was extensive neuronal loss and numerous vacuolated spaces, and intact neurons were absent. The corresponding area in aLA-treated animals exhibited partial neuronal loss and intact neurons were evident between vacuolated spaces. There was a restorative zone where the tissue had grown to invade the infarct core region. In addition, the number of processes was significantly increased in animals treated with aLA when compared with control animals. In the aLA group, nestin- and GFAP-positive cells were stellar in shape with large cell bodies and multiple processes when compared with the cells of control group, which had small cell bodies with marked vacuolization, degradation, and loss of processes. At 3, 7, and 14 days post-MCAO, IHC staining of brain sections revealed an increase in the number of nestin- and GFAP-positive cells in the aLA group when compared with the control group (Figures 5, 6 and 7).

### Neuroproliferation after aLA treatment

**Figure 8:**
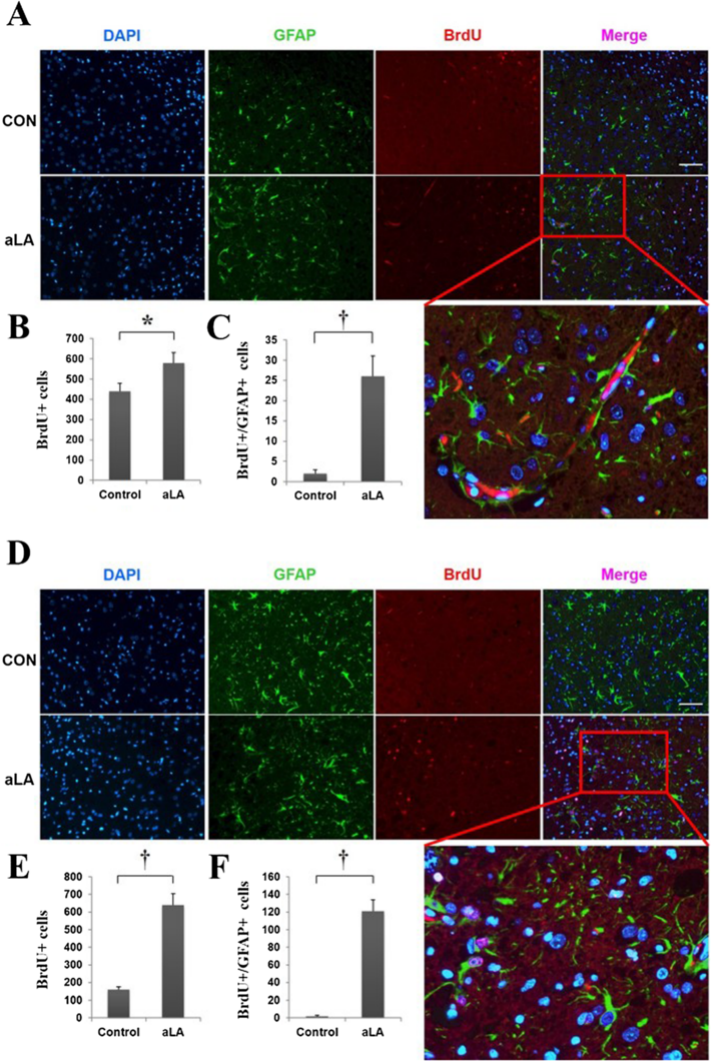
**Immunofluorescence for BrdU (red) and GFAP (green).** aLA increased the absolute number of newly developing astrocytes (BrdU/GFAP-positive) in the frontal and temporal boundary zone at 7 days after ischemia **(A and D)**. Bar graph showing the absolute numbers of newly developing glia (BrdU/GFAP-positive) in the brain (**B, C, E**, and **F**; *P < 0.05; †*P* < 0.01 when compared with the control group; Student’s unpaired *t* test). Data represents mean ± SEM (n = 4 rats/day/group). Scale bars = 100 μm.

**Figure 9:**
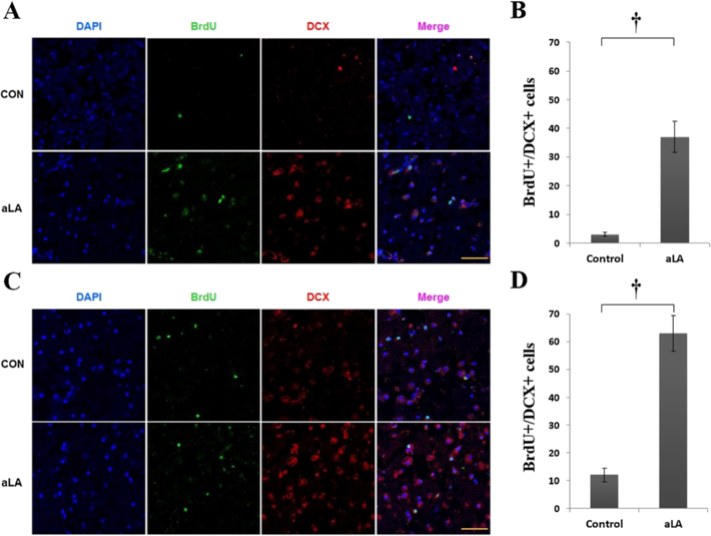
**Immunohistochemistry for DCX (red) and BrdU (green).** aLA treatment increased early neurogenesis (BrdU/DCX-positive) in the frontal **(A)** and temporal **(C)** boundary zone at 2 weeks after stroke. Bar graph showing the absolute numbers of co-labeled cells (BrdU/DCX-positive) in the brain (**B** and **D**; *P < 0.05; †*P* < 0.01 when compared with the control group; Student’s unpaired *t* test). Data represents mean ± SEM (n = 4 rats/day/group). Scale bars = 50 μm.

**Figure 10:**
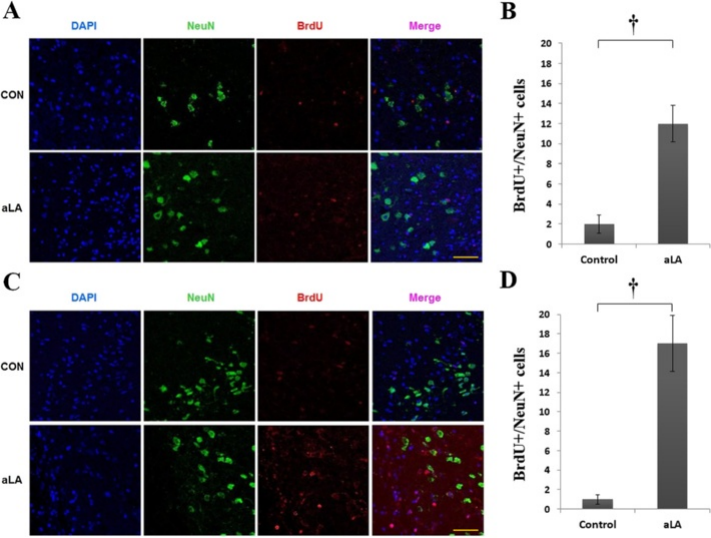
**Immunohistochemistry for BrdU (red) and NeuN (green).** aLA increased the co-localization of neurons and markers of proliferation in the frontal **(A)** and temporal **(C)** boundary zone at 4 weeks after ischemia. Bar graph showing the absolute number of newborn neurons (BrdU/NeuN-positive) in the brain (**B** and **D**; *P < 0.05 when compared with the control; Student’s unpaired *t* test). Data represents mean ± SEM (n = 4 rats/day/group). Scale bars = 50 μm.

To identify the cell lineage of proliferative cells, we performed double immunohistochemistry using BrdU, GFAP, DCX, and NeuN in frontal and temporal boundary zone (Figure 1) Immunochemistry against BrdU showed that aLA group had a greater number of BrdU-positive cells in the cerebral cortex (*P* < 0.001) than the control group (Figure 8). In addition, BrdU and GFAP double staining showed an increased astroglial proliferation (*P* < 0.001) in the aLA group compared with the control group (Figure 8). aLA-treated rats had a significantly higher number of BrdU/DCX-positive cells, currently a gold standard in measuring neurogenesis, in both the frontal and temporal boundary areas versus control rats 2 weeks after MCAO (Figure 9). The number of BrdU/DCX-positive cells in rats treated with aLA after brain ischemia was 5 fold more than that in vehicle-treated rats. To determine whether the early proliferative effects of aLA on post-stroke neurogenesis seen at 2 weeks were sustained, we evaluated the number of cells co-labeled with BrdU and the mature neuronal maker NeuN 4 weeks after stroke. aLA-treated rats showed an increased number of BrdU/NeuN-positive cells in the cerebral cortex (*P* < 0.001) when compared with control (Figure 10). Therefore, these results indicate that aLA strengthens intrinsic neurogenic processes as higher numbers of BrdU/GFAP, BrdU/DCX, and BrdU/NeuN-positive cells were found in the peri-infarct cortex.

### Effect of aLA treatment on inflammation and neurorestoration after ischemic insult

**Figure 11:**
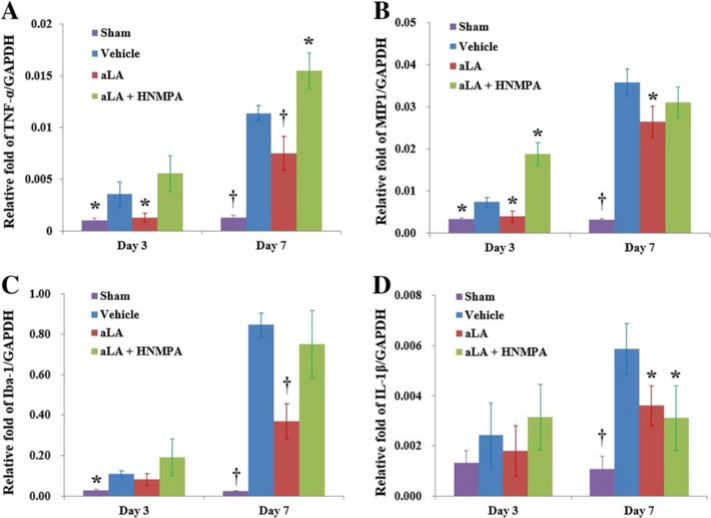
**The effects of aLA on the mRNA expression of inflammatory markers in the cortex of rats 3 and 7 days after cerebral ischemia, as measured by RT-PCR analysis. (A)** TNF-α mRNA expression. **(B)** MIP1 mRNA expression. **(C)** Iba-1 mRNA expression. **(D)** IL-1β mRNA expression. The expressions were normalized to glyceraldehyde 3-phosphate dehydrogenase (GAPDH) levels and expressed as relative fold activation. Data represents mean ± SEM (n = 3 rats/day in aLA, control, and HNMPA groups, n = 1 rat/day in the sham group). *P < 0.05; †*P* < 0.01 as compared to the control.

**Figure 12:**
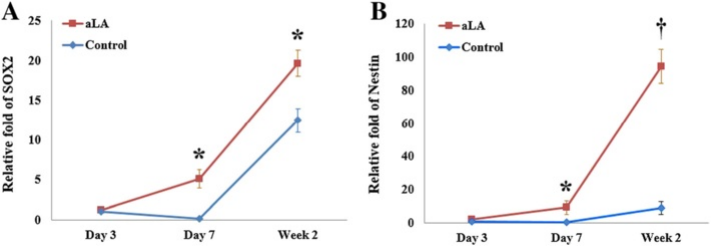
**Changes of relative mRNA expression of SOX2 and Nestin compared to the control group at3 days after cerebral ischemia, as measured by RT-PCR analysis. (A)** SOX2 mRNA expression. **(B)** Nestin mRNA expression. aLA increased the mRNA expression of SOX2 and Nestin at 7 and 14 days after ischemia. Data represents mean ± SEM (n = 3 rats/day in aLA and control groups, n = 1 rat/day in the sham group). *P < 0.05; †*P* < 0.01 when compared with the control group.

To analyze the basic mechanism and possible relationship between the neurorestorative effects of aLA and changes in inflammatory cytokine levels, TNF-α, MIP1, Iba-1, and IL-1β mRNA expression was determined by RT-PCR. Expression of TNF-α and MIP1 were significantly lower in the brains of aLA-treated rats than that in vehicle-treated rats 3 days after stroke (*P* < 0.05, Figure 11). The expression of TNF-α, MIP1, Iba-1, and IL-1β were significantly lower in the aLA group than in the control group at 7 days after stroke (*P* < 0.05, Figure 11). Employment of the IR inhibitor HNMPA(AM)3 completely inhibited aLA-induced anti-inflammatory properties. Conversely, coadministration of the HNMPA augmented the level of inflammatory cytokine induction such as TNF-α (Figure 11). Additionally, aLA increased the relative mRNA expression of SOX2 and nestin when compared with the control group at 3 days after stroke (*P* < 0.05, Figure 12).

## Discussion

We have shown that urgent treatment with aLA (20 mg/kg) after ischemic injury had long-term (56 days) neurorestorative effects against neural damage caused by cerebral infarction in rats. Furthermore, these long-term neurorestorative effects of aLA may be due, at least in part, to enhanced neuroproliferation and insulin receptor (IR) activation. Previous *in vivo* studies examining the neuroprotective effects of aLA have focused solely on its role in diminishing oxidative stress [11-15]. Given the increased use of aLA by the general population, the current results are of potential importance in the clinical setting.

Administration of aLA has been shown to reduce infarct size in several different models of MCAO in rodents [11-15]. These earlier studies assessed the effect of pretreatment with aLA, ranging from acute pre-administration to several daily injections for up to a week prior to MCAO, and survival times for periods of 1 to 7 days following MCAO. However, none of these studies demonstrated whether aLA is still effective if given after the onset of ischemia. In addition, the studies had limited duration, with post-ischemic evaluations over 7 days or less. Therefore, these models only represented events associated with early ischemic injury and did not address whether aLA has an effect later in recovery. Furthermore, each of these studies assessed neuroprotection at a single time point. For the first time, we report the long-term neurorestorative effects of urgent treatment with aLA after cerebral ischemia.

Treatment with aLA resulted in a 26% relative reduction in lesion size at 7 days. The magnitude of this reduction was similar to previous reports of reduced ischemic cell damage in MCAO rat models in response to various other agents [25-28]. Furthermore, the metabolic defect, as measured by ^18^F-FDG microPET imaging, in the cortex and striatum of the affected hemisphere significantly recovered after aLA treatment, while no recovery was seen in the control group, at 7 days after cerebral ischemia. Urgent treatment with aLA after cerebral ischemia was associated with improvement in functional outcomes after 56 days. The ultimate goal of therapy after stroke is improved functional outcome over time, the ability to measure improvements in functional deficits is essential for new therapeutic applications. In the current study, functional outcome was assessed by various neurobehavioral tests and was significantly improved in the aLA group when compared with the control group over a relatively long period of time (56 days).

The long-term neurorestorative effects of aLA were associated with several markers of neuroproliferation. This is a novel finding and demonstrates for the first time a putative mechanism of neurorestoration by aLA involving enhanced neuroproliferation. Both nestin and GFAP expression were significantly increased after treatment with aLA, based on IHC staining of brain sections. Nestin is a distinct intermediate filament protein that is transiently expressed in proliferating neuroepithelial stem cells during the neurulation stage of development.[29] Nestin expression has been used extensively as a marker for central nervous system (CNS) progenitor cells, as there is a correlation between nestin expression and this cell type *in vivo* [30]. Nestin has also recently been implicated as a novel angiogenesis marker for proliferating endothelial cells [31]. Following brain injury, increased nestin expression plays an important role in, and promotes the functional repair of, neuronal processes and synaptic plasticity [32].

Astrocytes increase following MCAO [33]. A growing body of data demonstrate that astrocytes respond to ischemia with functions important for neuroprotection and neurorestoration [34]. The rapidly expanding astrocytic processes create both physical and functional walls surrounding the ischemic core, which extend the time available for marshalling endogenous repair mechanisms [35]. Astrocytes are robustly immunoreactive for anti-oxidant proteins such as glutathione peroxidase, heme-oxygenase 1, and DJ-1 in infarcts; therefore, these glial cells are relatively resistant to oxidative stress compared to neurons, and important in neuronal antioxidant defense and secrete growth factors [36,37]. Furthermore, astrocytes also play an important role to promote neurorestoration in stroke [38]. Astrocytes effect long-term recovery after brain injury, through neurite outgrowth, synaptic plasticity, or neuron regeneration, and is also influenced by astrocyte surface molecule expression and trophic factor release [39-41]. In addition, the death or survival of astrocytes themselves may affect the ultimate clinical outcome and rehabilitation through effects on neurogenesis and synaptic reorganization [41]. Astrocyte cultures derived from the hippocampus have been shown to promote neurogenesis [42]. Astrocytes appear to play an important role in neurorestoration following CNS injury and promote neuronal differentiation [43]. It is clear that astrocytes are important in brain plasticity and recovery after stroke [44-46]. Astrocytes are characterized by a high level of GFAP expression, which has led to GFAP being one of the most frequently used astrocyte markers [47]. For these reasons, astrocytic activity, expressed by GFAP staining, reflects the outcome of the ischemic injury [48].

IHC staining using rat-specific anti-nestin and anti-GFAP antibodies show that aLA enhanced endogenous nestin and GFAP expression at 3, 7, and 14 days after MCAO. Nestin- and GFAP-positive cells were significantly increased with aLA treatment when compared with control animals in the peri-infarct and infarct core regions. Furthermore, the relative mRNA expression of nestin was significantly increased after aLA treatment when compared with control group at 3 days after ischemia. Additionally, the morphological features of nestin- and GFAP-expressing cells, such as the number and shape of cell bodies and processes, were significantly improved in the aLA group. These results suggest that aLA treatment increases the number of nestin- and GFAP-expressing cells, showing a marked neuroproliferative effect following ischemic brain damage. These results are consistent with an earlier study of cultured astroglial cells, which showed that nestin and GFAP expression increases significantly after aLA treatment [16]. In this study, the proliferation and differentiation of astrocytes in culture correlated with the antioxidant properties of aLA, particularly with its ability to restore glutathione content. These findings have led to the hypothesis that the neurorestorative effects of aLA against oxidative stress promote the proliferation and differentiation of astroglial cells.

Most importantly, there was a restorative zone shown in the IHC and immunofluorescence staining, where the cells had grown into the infarct core region from the boundary of the lesion after aLA treatment. aLA significantly increased the proliferating astrocytes (BrdU/GFAP-positive) in the boundary zone at 7 days after ischemia in line with IHC. In ^18^F-FDG microPET imaging, the brain metabolism in the BrdU/GFAP-positive boundary zone at 7 days recovered in the aLA group. In the previous studies, the majority of ^18^F-FDG hyperuptake regions were not recruited in the final infarction, suggesting that the ^18^F-FDG uptake may be associated with neuronal survival and activity [49-51]. The increased ^18^F-FDG uptake might be through facilitative expressing of GFAP-positive cells [52,53]. These results suggest that BrdU/GFAP positive cells might promote neuronal survival and neurorestoration.

Moreover, aLA treatment increased BrdU/DCX co-labeled cells, a marker for early neurogenesis, in the boundary zone at 2 weeks after stroke. By 4 weeks after stroke, BrdU positive cells developed into newborn neurons (BrdU/NeuN-positive), suggesting that these cells had survived, and the early proliferative effects of aLA on post-stroke neurogenesis seen at 2 weeks were sustained.

The mechanism by which aLA promotes neuroproliferation could be related to the anti-inflammatory and anti-oxidant properties of aLA. It has been suggested that reactive oxygen species inhibit the actions of stem cells and that suppressing oxidative stress promotes a restorative effect of stem cells against ischemic injury [54-56]. Antioxidants also promote growth of progenitor cells and have been shown to play a role in the growth, development, protection of stem cells [54,57]. In our study, aLA treatment significantly decreased the expression of major inflammatory cytokines that play an important role during the acute phase of stroke such as TNF-α, MIP1, Iba-1, and IL-1β in RT-PCR. In addition, aLA significantly increased the mRNA expression of SOX2, a transcription factor that is essential for maintaining the self-renewal properties, or pluripotency, of undifferentiated embryonic stem cells. These mechanisms may play a pivotal role in neurorestoration following cerebral ischemia, further supporting the utility of this antioxidant as a neurorestorative agent.

IR inhibitor HNMPA(AM)3 has been evaluated in recent studies to demonstrate IR dependent effects [17,18]. The previous study demonstrated a direct binding site for aLA to the tyrosine kinase domain of the IR, and HNMPA(AM)3 blocked the protective effects of aLA for apoptosis [17]. aLA also has been reported to directly activate dopamine receptor and peroxisome proliferator activated receptor gamma (PRAR-γ) [58,59]. To verify the effect of aLA on cerebral ischemia, we tested the effects of aLA via IR could be blocked by HNMPA(AM)3 or not. Consequently, pretreatment of IR inhibitor blocked aLA-induced neuroprotection and functional recovery. Inflammatory cytokine levels were markedly increased in the HNMPA group compared to aLA group. These results suggest that the effects of aLA are mediated at least partially via IR activation, a well-documented neuroprotective pathway in ischemic models [60-62]. However, more research is needed to determine the underlying mechanisms and/or link between neuroproliferation and aLA in cerebral ischemia.

There were some important limitations to this study. We used young rats rather than older animals. Young animals may easily enhance neurogenesis after stroke compared to aging animals. Moreover, markers for neurogenesis and inflammatory cytokines are known to change with age. Therefore, this could be the major limitation of our work for translation into the clinic. Another limitation of our study is that we did not investigate the effects of different (i.e., increased) doses or repeated infusions of aLA. Instead, we selected the most suitable dose of aLA based on currently available literature.

In conclusion, aLA acts as a potent neuroprotectant by promoting neuroproliferation following ischemic brain damage. Despite sophisticated medical management and the availability of neurosurgical techniques, MCA territory infarction still results in a high mortality rate. Therefore, neurorestorative and survival pathways represent potential therapeutic targets for acute ischemic injury in clinical settings. The current study is the first to demonstrate that urgent aLA treatment given post-stroke within a short treatment window has a significant neurorestorative effect and promotes long-term functional recovery through enhanced anti-inflammatory and anti-oxidant actions mediated at least partially via IR activation. These results provide further evidence of the positive therapeutic effects of aLA in the treatment of ischemic stroke and allow developing novel clinical approaches to minimize ischemia/reperfusion damage.
